# Postoperative nausea and vomiting in a gynecological and obstetrical population in South Eastern Nigeria

**DOI:** 10.4314/pamj.v7i1.69111

**Published:** 2010-10-19

**Authors:** Ugochukwu Okafor, Adaobi Amucheazi, Richard Ewah, Okezie Obioma

**Affiliations:** 1 Department of Anesthesia, University of Nigeria, Enugu Campus, Enugu, Nigeria/ Department of Anesthesia, University of Nigeria Teaching Hospital (UNTH), Ituku Ozalla, Enugu, Nigeria; 2 Department of Obstetrics and Gynecology, University of Nigeria teaching hospital (UNTH), Ituku Ozalla, Enugu, Nigeria.

**Keywords:** Postoperative, nausea, vomiting, obstetrics and gynaecology, Africa, ethnicity

## Abstract

**Background:**

To determine the incidence of Postoperative nausea and vomiting (PONV) in a high risk surgical group following studies in other predominately black populations that showed a lower rate of postoperative nausea and vomiting than that reported from Caucasian and Oriental populations.

**Methods:**

A retrospective observational survey was conducted in the University of Nigeria Teaching Hospital (UNTH), Enugu, Nigeria to determine the incidence of PONV within forty-eight hours of anesthesia in an obstetrical (caesarean sections only) and gynecological population that underwent regional and general anesthesia. The study took place from December 2007 – April 2009 (16 months) for the gynecological population and from May 2008 to May 2010 (25 months) for the obstetrical population. The folders of 300 patients were randomly reviewed for demographics, anesthetic technique, diagnosis and documented records of PONV within 48hours of anesthesia.

**Results:**

A total of 300 obstetrical and gynecological patients were used in this study. Twelve women vomited within forty-eight hours of anesthesia (12/300 or 4.0%). Nine patients vomited in the gynecological population (9/112) or 8% of the gynecological population and (3/186) or 1.6% in the obstetric population. All patients were American Society of Anesthesiologists (ASA) 1-4 including surgical emergencies.

**Conclusion:**

The incidence of PONV in this surgical population is lower than that from most of the studies reviewed. This might be due to an inherent ethnic/racial variation. The economic implication of spending on expensive anti-emetics means more money can diverted to other needs.

## Background

Postoperative nausea and vomiting (PONV) is a common complication of anesthesia and surgery. It is considered the most common cause of morbidity following anesthesia [1-4] and has significant effects on patient satisfaction and cost [3,5].

Despite advances in anti-emetic therapy, the incidence of PONV is reportedly 20 – 30% and increases to about 70% of those with certain risk factors [6]. These risk factors include gender (female sex) and breast, middle ear, gynecological and obstetrical surgery [7-9]. Amongst other possible risk factors for PONV is ethnicity and genetics. The incidence in the obstetric and gynecologic population reportedly ranges from 40% - 80% [7-9]. This has great psychological, physical, emotional and financial consequences because of the distress caused by the vomiting including electrolyte imbalance, wound dehiscence and the extra cost incurred because of delayed discharge. Consequently, a lot of resources have been expended in coming up with a potent antiemetic that would greatly reduce the incidence of PONV in patients.

Some studies in Black populations have reported comparatively lower figures of PONV [10-12], suggesting the possible role of pharmacogenomics in obstetric care. It is important to establish whether some Blacks population in Africa and the diaspora are less prone to PONV than the other races and the possible reasons for that. Nigeria is the most populous Black nation in the world with an estimated population of 149 million [13]. This study was thus undertaken to determine the incidence of PONV in a high risk surgical population and to elucidate whether such a trends exists amongst the Igbos (18% or 26 million of Nigeria’s population) of South – Eastern Nigeria [13].

## Methods

A retrospective observational survey was conducted in the University of Nigeria Teaching Hospital (UNTH), Enugu, Nigeria to determine the incidence of PONV within forty-eight hours of anesthesia in an obstetrical (caesarean sections only) and gynecological population that underwent regional and general anesthesia.

The study took place from December 2007 to April 2009 (16 months) for the gynecological population and from May 2008 to May 2010 (25 months) for the obstetrical population.

A total of 529 obstetrical and gynecological patients underwent anesthesia (147 for the gynecological group and 382 for the obstetrical group) during the study period. The Gpower 3.1.0 software was used to determine the sample size using a power of 80, a prevalence rate of 25 percent, a confidence level of 95% and a finite population of zero (to increase the sample size) to give a required sample size of 289.

There were 112 gynecological patients and 188 obstetric patients making for a total of 300 patients. The folders of the 300 patients were reviewed for demographics, anesthetic technique, diagnosis and documented records of PONV within 48 hours of anesthesia. Being a retrospective study, the incidence of nausea may not have been reliably documented and only those that had resultant therapeutic intervention as documented in their folders were used in this study.

Nausea is the subjective feeling of a need to vomit. Autonomic symptoms such as pallor, tachycardia, diaphoresis, and salivation often accompany this feeling [14]. Vomiting is a reflexive, rapid, and forceful oral expulsion of upper gastro-intestinal (GI) tract contents due to powerful and sustained contractions in the abdominal and thoracic musculature [15].

The hospital protocol for neuraxial blocks involves premedication with gastric prokinetic agents and H2 receptor antagonists and for operations under general anesthesia, the anti-cholinergics atropine and glycopyrollate. The popular analgesics are ketamine, tramadol and the opiates fentanyl, pethidine and pentazocine. The volatile agents are halothane and isoflurane. Nitrous oxide is expensive in Nigeria and medical air and Page number not for citation purposes 3 oxygen has been used in our centre for more than 6 years. The anti-emetics used in our centre for surgical patients are metoclopramide and promethazine. Pethidine, pentazocine and tramadol are the postoperative analgesics of choice. In our centre, postoperative patients are first taken to the recovery room, where their vital signs and arterial oxygen saturation are monitored until they recover from anesthesia (with stable vital signs) and are then transferred to their respective wards. Cardiac and critically ill patients are usually taken to the intensive care unit (ICU) for postoperative care. In early 2007, the hospital moved to its permanent site where the theatre and recovery room or post-anesthetic Care unit (PACU) was well equipped by VAMED health care services, an Austrian health care service provider. They installed multi-channel monitors (pulse oximetry, non-invasive blood pressure monitor, temperature, electrocardiography and capnography), and modern anesthetic machines with low flow systems.

Ethical board approval is not mandatory for medical audits/retrospective studies.

## Results

A total of 300 obstetrical and gynecological patients were included in this study. A total of 12 women vomited within forty-eight hours of anesthesia (12/300 or 4.0%, Table 1).

Nine patients vomited in the gynecological population (9/112) or 8% of the gynecological population and (3/188) or 1.6% in the obstetric population. In the obstetric population, 165 parturient delivered under regional anesthesia, while 23 delivered under general anesthesia. In the gynecological group there were nine regional techniques and 103 received general anesthesia.

Sixty-three (56%) of the 112 gynecological patients received intraoperative opioids and less than a third of the caesarean deliveries received intraoperative opioids including intrathecal opioids. All the gynecological patients that had PONV received general anesthesia; while the caesarean section patients all delivered under spinal subarachnoid anesthesia with opioid supplementation in two patients (all three were morbidly obese). All patients were American Society of Anesthesiologists (ASA) 1-4 including surgical emergencies. There were no laparoscopic surgeries. There were ten documented day cases in the gynecological population who were discharged on oral non-opiates.

## Discussion

The incidence of PONV in this study is quite low compared to some studies from most regions of the world [7-9,16] including Asia, Europe and North America. This is despite the fact that a significant number of these patients received opioids peri-operatively. Though anticholinergics like atropine and glycopyrrolate and the anti-emetic, metoclopramide were premedicants in this study and nitrous oxide was not used due to unavailability, the figures were lower than those in surgical populations that received preoperative potent anti-emetics like the serotonin antagonists.

The low incidence of PONV in our surgical population has been noted for some time, which is probably why potent anti-emetics were not in use in our theatres; the first line anti-emetics being promethazine and metoclopramide. This is significant because the pharmacy stocks expensive anesthetics like propofol and isoflurane. Studies in high risk surgical populations in predominantly black West Indies have revealed similar trends [11-12]. One explanation could be an intrinsic variation between the races.

Harvey [12] reported an overall incidence of 2.9% PONV in the recovery room in Guyana, which was significantly lower than that reported among Caucasian populations. A study from Nigeria also reported a low incidence of vomiting (1%) in the recovery room and 19.6% for the first 24 hours postoperatively [10]. The surprising thing is that our figures are even lower than those from other Nigerian studies which reported incidences of Page number not for citation purposes 4 about 20% in their studies [10,17]. This may signify that even amongst the same race, there could be variations, though the fact that we rarely use nitrous oxide which is synergistic with opioids in causing PONV might be the reason for the disparity [15].

The import of this study may be two-fold; namely the economic benefits of the low incidence of PONV and the possible underestimated value of atropine as a potent anti-emetic. Our patients receive anticholinergics to dry secretions caused by ketamine which is popular as an effective analgesic without respiratory depressant effects. Caesarean section patients’ received metoclopramide which is a gastric prokinetic agent used to speed up gastric emptying.

Anticholinergics block cholinergic central nervous system emetic receptors in the cerebral cortex and pons [18]. Their addition to opioid premedication is known to reduce emesis [19,20]. Though dietary supplements/spices like ginger and red pepper have been mentioned, ginger is not part of the local diet. Pepper is an important part of the local diet, and maybe that route could be explored.

In a study of 2722 patients, Apfel et al developed a simplified risk score consisting of four predictors for PONV; female gender, history of motion sickness or PONV, non-smoking status and the use of opioids for postoperative analgesia. In their study if none, one, two, three or four of these risk factors were present, the incidences of PONV were 10, 21, 39, 61 and 79% respectively [21].

Almost all our patients had three risk factors; female gender, non-smoking status and postoperative opioid use.

In economic terms, the cost of Ondansetron 4mg is about 22 United States Dollars (USD). Since a reported majority of Nigerians live on a dollar a day, hospital budgets can be used to accommodate more pressing needs like potent parenteral antibiotics etc.

Another important economic factor is that the routine use of the expensive nitrous oxide for general anesthesia is unnecessary. It is known to increase the incidence of PONV by as the cost of anesthesia for the patients [5,22,23].

A randomized prospective study to determine the role of atropine in reducing PONV is to start in South-Eastern Nigeria soon. We hope that the study will shed more light on what is essentially a hypothesis; that some ethnic groups are less prone to PONV and atropine is an effective antiemetic.

An interesting study from Norway of 900,000 first time pregnancies over a 40 year span showed significant variations in the incidence of hyperemesis gravidarum amongst the various ethnic groups in the country. Mothers born in India and Sri Lanka (3.2%) had the highest prevalence of hyperemesis gravidarum, followed by those born in Africa (excluding North Africa) at 3.1% and Pakistan 2.1% [24]. Ethnic Norwegians, North Americans and Western Europeans had the lowest prevalence of 0.9 percent, 0.9% and 0.8%, respectively [24]. Limitations in this study include the retrospective nature, possible sampling bias and a lack of knowledge of the patients’ history of PONV.

## Conclusion

The incidence of PONV in this surgical population is lower than that from most of the studies reviewed. This might be due to an inherent ethnic/racial variation. The economic implication of spending less on expensive anti-emetics means more money can be diverted to other needs. The non-use of nitrous oxide in this population may have contributed to these low figures.

## Competing interests

There are no competing interests to declare.

## Authors’ contributions

UVO and OO collected the data; UVO analysed it with intellectual input from AA and RE.

## Figures and Tables

**Table 1:** Total number of surgeries, anesthetics and postoperative nausea and vomiting

**Total number of patients (n=300)**	**Caesarean deliveries (n=188)**	**Gynecological patients (112)**	**Total (%)**
Postoperative nausea and vomiting (n=12)	3 (1.6%)	9 (8%)	12 (4%)
General anesthesia (GA)	23 (13%)	103 (92%)	126 (42%)
Regional anesthesia (spinal and epidural) (RA)	165 (87%)	9 (8%)	174 (58%)
Relationship of postoperative nausea and vomiting to anesthetic technique N=12	RA-3, GA-0	GA-9, RA-0	GA-9, RA-3
Day cases	0	12	12
